# Constitutions of Deficiency and Stasis of Traditional Chinese Medicine and Related Factors among Middle-Aged Women in Taiwan

**DOI:** 10.1155/2020/7237029

**Published:** 2020-10-19

**Authors:** Ping-Ho Chen, Sheng-Miauh Huang, Jerry Cheng-Yen Lai, Pei-Jung Yu

**Affiliations:** ^1^Department of Traditional Chinese Medicine, Taipei Medical University Hospital, Taipei, Taiwan; ^2^Department of Nursing, Mackay Medical College, New Taipei City, Taiwan; ^3^Department of Medical Research, Taitung MacKay Memorial Hospital, Taitung, Taiwan

## Abstract

**Background:**

Traditional Chinese medicine (TCM) appears to be the common therapy in middle-aged women. The constitution serves as a guide for TCM treatment. However, little is known about the constitution and related factors in middle-aged women. The objectives of this study were to describe the yang-deficiency, yin-deficiency, and stasis constitutions in middle-aged women. Demographic and health factors related to yang deficiency, yin deficiency, and stasis were also examined.

**Methods:**

A total of 1,000 women aged 40–65 years were selected from 2009 through 2018 using random sampling from the Taiwan Biobank Research Database in Taiwan. Yang-deficiency, yin-deficiency, and stasis were assessed using the body constitution questionnaire. Multiple logistic regression analysis was used to identify factors associated with constitution in deficiency or stasis.

**Results:**

The proportions of middle-aged women who had the constitution in yang-deficiency, yin-deficiency, and stasis were 29.7%, 21.7%, and 17.7%, respectively. The result of binary logistic regression showed that current menstruation, abnormal spirometry, and education level were predictive factors of yang deficiency. Women with younger age, abnormal spirometry, or a vegetarian diet had a significantly associated yin deficiency. Younger age, abnormal spirometry, and coffee habit were predictors of stasis.

**Conclusions:**

Middle-aged women in Taiwan with abnormal spirometry had a higher risk for deficiency or stasis constitutions, especially for those younger than 56 years. Healthcare providers should learn patients' constitutions and provide appropriate advice, referring them to safe providers of their desired method.

## 1. Introduction 

The identification of constitution, a concept that has been developed in the 1970s in China, provides basic guidance in the traditional Chinese medicine (TCM) diagnostic and therapeutic systems. A constitution can be viewed as the individual's body condition that makes an individual susceptible to certain diseases but not others. The literatures indicate patients with different constitutions are treated differently [1–3]. The basis of the yin-yang theory in TCM is that yin and yang are the two opposite, complementary, and interrelated cosmic forces found in all matter in nature. All changes that are observed in the world, including those of the human body, arise from the ceaseless motion of both yin and yang. If yin-yang has deviated or is out of balance, some psychophysiological symptoms and patterns appear, such as deficiency and stasis [4, 5]. The deficiency in constitutions include yang deficiency and yin deficiency. The former is marked by a low energy (qi) level in the physiological functioning of the body. The latter implies that a person has insufficient yin fluid [4–6]. A stasis indicates that the dynamic yin and yang interactions are slowed or deﬁcient, which usually happens in patients with a disease because their qi and blood are obstructed by the lesion site, which is prevalent among postmenopausal women [4–7]. It is believed that a patient may experience different constitutions at the same time, and the constitution is dynamically changing with pathological, physiological, and psychological states [8, 9].

In 2015, the World Health Organization released the ﬁrst world report on healthy aging [10]. There is increasing interest in the clustering of risk behaviors in elderly people. However, identifying leading predictors for disease-free status in the middle-age group may provide evidence for action priorities in elderly care. [11, 12]. Middle-aged women experience many physical and mental changes. Previous studies have linked socioeconomic status and lifestyle, behavioral, psychological, and biological factors to healthy aging [13–15]. Body mass index and diet behavior are key factors for disease-free status promotion [11,12,14]. Women living with climacteric symptoms experienced negative physical and psychological consequences that led to changes in their lives, particularly in their relationships [16]. A systematic review shows that menopause is a stage of life experienced in different ways, characterized by personal challenges and changes in personal roles within the family and society. Those women reported hot flushes and night sweats are the strongest symptoms during menopause [17]. Osteoporosis may affect one out of three postmenopausal women. The risk of osteoporosis and fragility fractures can be reduced through healthy lifestyle changes [18, 19]. Chronic obstructive pulmonary disease and airway obstruction were also related to bone mineral density in postmenopausal women [20]. Those symptoms were consistent with the performance of constitutions in deficiency and stasis [7, 21]. No study has examined the relationship between health indicators and constitutions.

Both modern medicine and TCM expenses are covered in parallel by the National Health Insurance scheme in Taiwan. More than one-quarter of the people have ever used Chinese medicine in Taiwan, especially women, and this is expected to continue in the future [22–24]. TCM might have a positive impact on the relief of climacteric symptoms and the prevention of osteoporotic fracture [25, 26]. TCM doctors evaluated periodical treatment as an important part of the process of individual treatment based on differentiation of constitution. [27, 28]. No major studies have measured the constitution in middle-age women in Taiwan. Therefore, we have little knowledge of the constitution differences between the deficiency (yin and yang) and stasis. No study has examined the health determinants of constitution. Therefore, the objectives of this study were to describe constitutions in yang deficiency, yin deficiency, and stasis among middle-aged women. In addition, demographic and health factors related to yang deficiency, yin deficiency, and stasis were examined. The results could allow TCM doctors to plan health promotion programs to ﬁt the demands of TCM and personal needs in the follow-up with middle-age women.

## 2. Materials and Methods

This study was based on the analysis of secondary data. All data were provided from the Taiwan Biobank, which collected specimens and relevant information from individuals in recruitment centers across Taiwan. The study was approved by the human subjects committee at MacKay Memorial Hospital in Taipei. All participant names were replaced by coded numbers to ensure anonymity. Written consent was not required since patient identifiers were not included in the data.

### 2.1. Study Participants

Middle age, as defined in the Oxford Learners Dictionary, is the period between early adulthood and old age, usually considered as the years from about 45 to 60 [29]. The Merriam–Webster Dictionary defines middle age from about 40 to 64 years [30], whereas the prominent psychologist Erik Erikson saw it as starting a little earlier and defines middle adulthood as between 40 and 65 years [31]. Hence, the inclusion criteria in our study were women: (1) age, 40–65 years; (2) study period, 2009 to 2018; (3) completed the survey including diet behavior, female characteristics, health examination, and constitutions. The sample size was estimated using *G*^*∗*^ Power: Statistical Power Analyses software. Logistic regression was used to compute the required sample size. The *α* error probability was set to 0.05; the power was 0.95, and the odds ratio was set at 1.3. The calculated sample size was 988. Among the 27,366 potential study candidates, a total of 1,000 women aged 40–65 years were selected from 2009 to 2018 using random sampling from the Taiwan Biobank Research Database. Taiwan Biobank aims to build a nationwide research database that conducts large-scale cohort studies and case-control studies on local diseases. About 200,000 volunteers will be called for in the cohort study, while 100,000 patients with the 10 to 15 most common diseases will be invited in the case-control study.

### 2.2. Measurements

The study variables included demographics (age, marital status, work status, and education), dietary behavior (vegetarian, tea habit, and coffee habit), female characteristics (menstruation, pregnancy, and abortion), health examination (body mass index, waist and hip circumference, bone mineral density, and pulmonary function), and constitutions (yang deficiency, yin deficiency, and stasis).

The Body Constitution Questionnaire (BCQ) was developed to measure psychological and physiological states of deviation in the constitution. The BCQ instrument was developed through a literature review and a Delphi process by 26 experts [32]. The reliability and validity of the scale were confirmed [33–36]. The BCQ included 44 items covering 3 identifications of the constitution: yang deficiency (19 items), yin deficiency (19 items), and stasis (16 items) (see the appendix). Each item was rated on a 5-point frequency or intensity Likert scale, ranging from 1 (never or not at all) to 5 (always or very severe). Assessments of internal validity, internal consistency, test-retest, and criterion-related tests were used to conﬁrm the reliability and validity of the BCQ [30–33].

A yang-deficiency constitution indicates that a person's energy (qi) for maintaining body functions is insufficient, and they may experience symptoms such as fatigue, shortness of breath, chills, loose stool, and a large volume of urine. A person was considered to have a yang-deficiency constitution if the sum of the BCQ + scores was ≥30.5 [32,33].

A yin-deficiency constitution means the insufficiency of body fluid, which means a person may present symptoms such as thirst, constipation, oral ulcers, and dry eyes. A total BCQ− score ≥29.5 was considered a yin-deficiency constitution [34]. The stasis constitution features the imbalance and obstruction of qi and blood. People with the stasis may have lesions of pain, numb limbs, sputum, or edema. A patient with a sum of BCQs scores ≥26.5 was considered to have a stasis constitution [35].

The analyzed data included the health examination results obtained for body mass index, waist and hip circumference, bone mineral density, and pulmonary function. Body mass index was calculated as a person's weight in kilograms divided by the square of height in meters. Categories of body mass index included underweight (under 18.5 kg/m^2^), normal weight (18.5 to 25 kg/m^2^), overweight (25 to 30 kg/m^2^), and obese (over 30 kg/m^2^). The bone mineral density (BMD) *T* score is used to compare with the healthy young adult (average 30 years old), while the *Z* score compares an individual's bone density to the average bone density of people her own age and gender. A *T* (or *Z*) score within 1 means the individual's BMD is healthy. People with *T* (or *Z*) score between −1.0 and −2.5 means low bone density or osteopenia. A score lower than −2.5 is a diagnosis of osteoporosis. The results of pulmonary function tests including forced vital capacity (FVC), forced expiratory volume in one second (FEV1), FEV1/FVC ratio, and total lung capacity (TLC) are the indicators to evaluate the obstructive impairment, restrictive impairment, or mixed impairment. Women with normal FVC (≥80%), FEV1 (≥80%), and FEV1/FVC ratio (within 5% of the predicted ratio), and TLC (≥80%) were identified as a normal group of the pulmonary function test in our study.

### 2.3. Data Analysis

Statistical analyses were performed using the Predictive Analytics Suite workstation running version 18.0 software (PASW, IBM Corp., Somers, New York, USA). Individual variables were examined by percentages, means, and standard deviations. The chi-square test and *t*-test were used to determine the significance of differences in characteristics between the women who had deficiency or stasis constitution or not. We used multiple logistic regression analysis to identify factors associated with deficiencies or stasis constitutions. In all analyses, *P* < 0.05 was considered statistically significant.

## 3. Results

### 3.1. Characteristics of the Participants

The mean age of the study women was 51.98 years (SD = 6.84). More than half of the women were employed (58.1%), married (74.5%), and had been pregnant (88.9%). Some women had a habit of drinking tea (29.1%) or coffee (33.9%). Only 77 (7.7%) women were vegetarian. The number of women with current menstruation in age < 51 (years), 51 ≤ age < 56 (years), and 56 ≤ age < 65 (years) groups were 362 (86.4%), 79 (33.3%), and 4 (1.2%). The percentage of middle-aged women who had the constitution in yang-deficiency, yin-deficiency, and stasis was 29.7% (*n* = 297), 21.7% (*n* = 217), and 17.7% (*n* = 177), respectively (Table 1). Of the 356 women with one constitution at least, 35.1% (*n* = 125) women experienced yang-deficiency, yin-deficiency, and stasis constitutions at the same time, 23.8% (*n* = 85) women had two of three constitutions, and 41.0% women (*n* = 146) had one of three constitutions.

Among the women, 384 (38.4%) reported being overweight (24.4%) or obese (14%). The overall mean waist and hip circumference of the women were 80.88 cm (SD = 8.76) and 95.26 cm (SD = 6.92). The *Z* score for bone mineral density indicated 91 women had osteopenia or osteoporosis. The percentages of normal spirometry, obstructive impairment, restrictive impairment, and mixed impairment were 60% (*n* = 600), 22%, (*n* = 220) 13.7% (*n* = 137), and 4.3% (*n* = 43), respectively (Table 1).

### 3.2. Factors Related to Yang-Deficiency Constitution

Bivariate analyses showed that age, marital status, education, coffee habit, current menstruation, ever having been pregnant, and abnormal spirometry were significantly associated with the constitution of yang deficiency (Table 1). We included and adjusted for those variables in the multiple logistic regression analyses. Because the results of logistic regression with forward and backward methods showed no significant differences in age, marital status, coffee habit, and ever having been pregnant, we excluded those variables. Considering the possible interaction of age and menstruation status on yang deficiency, the data were divided by age groups to evaluate the menstruation effect on the yang-deficiency constitution. Among the three groups of age, there was no significant difference in the yang-deficiency constitution between women with and without menstruation (Table 2). The final model shows that women with abnormal spirometry had a higher risk for the yang-deficiency constitution (OR = 1.42, 95% CI 1.07–1.88, *P*=0.02). Participants with current menstruation had a greater risk for a yang-deficiency constitution (OR = 1.45, 95% CI 1.10–1.93, *P* < 0.01). Compared with the people with elementary-school education, those graduated from high school, university, or graduate school had a greater risk for a yang-deficiency constitution (high school: OR = 2.42, 95% CI 1.11–5.27, *P*=0.03; college or university: OR = 2.80, 95% CI 1.28–6.12, *P*=0.01; graduate school: OR = 2.75, 95% CI 1.08–6.97, *P*=0.03).  Table 3 presents the logistic regression models of the yang-deficiency constitution.

### 3.3. Factors Related to Yin-Deficiency Constitution

Bivariate analyses showed that age, vegetarian diet, coffee habit, current menstruation, bone mineral density *z* score, and abnormal spirometry were significantly associated with the constitution of yin deficiency (Table 1). We included and adjusted for those variables in the multiple logistic regression analyses. Because the results of logistic regression with forward and backward methods showed no significant differences for coffee habit, current menstruation, and bone mineral density *z* score, we excluded those variables. Considering the possible interaction of age and menstruation status on yin deficiency, the data were divided by age groups to evaluate the menstruation effect on the yin-deficiency constitution. Among the three groups of age, there was no significant difference in the yin-deficiency constitution between women with and without menstruation (Table 2). The final model shows that women younger than 56 years had a higher risk of yin-deficiency constitution (40 ≤ Age < 51: OR = 1.81, 95% CI 1.25–2.62, *P* < 0.01; 51 ≤ Age < 56: OR = 1.80, 95% CI 1.19–2.72, *P* < 0.01). Participants with abnormal spirometry also had a greater risk for the yin-deficiency constitution (OR = 1.48, 95% CI 1.08–2.01, *P*=0.01). Vegetarians in our study had a lower risk for the yin-deficiency constitution (OR = 0.28, 95% CI 0.12–0.65, *P* < 0.01).  Table 3 presents the logistic regression models of the yin-deficiency constitution.

### 3.4. Factors Related to Stasis Constitution

Bivariate analyses showed that age, marital status, currently working, coffee habit, current menstruation, and abnormal spirometry were significantly associated with the stasis constitution (Table 1). We included and adjusted for those variables in the multiple logistic regression analyses. Because the results of logistic regression with forward and backward methods showed no significant differences in marital status, current menstruation, and currently working, we excluded those variables. Considering the possible interaction of age and menstruation status on stasis, the data were divided by age groups to evaluate the menstruation effect on the stasis constitution. Among the three groups of age, there was no significant difference in stasis constitution between women with and without menstruation (Table 2). The final model shows that women younger than 56 years had a higher risk for a stasis constitution (40 ≤ Age < 51: OR = 3.08, 95% CI 2.00–4.75, *P* < 0.01; 51 ≤ Age < 56: OR = 2.29, 95% CI 1.41–3.74, *P* < 0.01). Participants with abnormal spirometry also had a greater risk for stasis constitution (OR = 1.63, 95% CI 1.16–2.28, *P* < 0.01). There was a greater risk for a stasis constitution in women with a coffee habit (OR = 1.49, 95% CI 1.06–2.08, *P*=0.02).  Table 3 presents the logistic regression models of the stasis constitution.

## 4. Discussion

We found that the yang-deficiency, yin-deficiency, and stasis constitutions were common in the middle-aged women, especially the yang-deficiency constitution. Of all middle-aged women, one in five suffered at least two constitutions at the same time. The result is consistent with TCM theory [1–3]. The simultaneous presence of two constitutions in an individual is like the concept of comorbidity in modern medicine. A constitution may include several diseases, whereas a disease can be expressed in different syndromes. As a disease advances, constitutions may transform into different zhengs [3, 37]. TCM doctors should be aware of the cooccurrence of deficiency and stasis among middle-aged women and focus their treatment on restoring balance to support their health.

We found that middle-aged women with current menstruation had deteriorating yang-deficiency constitution, whereas women younger than 56 years experienced worse yin-deficiency and stasis constitutions. This result is consistent with a previous study, which indicated the yin-deficiency, yang-deficiency, and stasis constitutions were significantly worse in perimenopausal and postmenopausal than premenopausal women with climacteric syndrome [7]. More than 21% of the climacteric women had ever visited TCM doctors [38]. *Eclipta prostrata* with *Ligustrum lucidum* and dan-zhi-xiao-yao-san were the most commonly used combination of Chinese herbal products by TCM doctors in Taiwan [39]. Nonetheless, more study is needed to examine the effectiveness of TCM treatment, such as scrapping, acupuncture, or herbal therapies, based on constitution classification among women with climacteric syndrome [39–41].

Drinking four or more cups of coffee per day is associated with a higher hip fracture risk, whereas moderate intake may alleviate the risk in postmenopausal women [42]. In our study, a coffee habit was also associated with an increased stasis constitution among the study participants. The stasis constitution manifests itself as fatigue, headache with pressure, or pain. Those symptoms could be associated with the motivation for drinking coffee. TCM doctors are expected to assess the causality between coffee drinking and stasis constitution. Suggesting a moderate intake of coffee might help reduce the stasis constitution and avoid worsening osteoporosis. In the study, osteoporosis was not significantly related to deficiency and stasis constitutions, which could be due to the small number of patients with osteoporosis. Besides, patients with osteoporosis tended to take more supplements. Future study is needed to examine the effectiveness of TCM treatment on decreasing other constitutions among patients with osteoporosis.

We found that women with abnormal spirometry had deteriorating the constitutions in yin-deficiency, yang-deficiency, and stasis. Some evidence indicates that TCM treatment could help alleviate pulmonary disease [43–45]. For example, the active agents of single herbs and TCM formulas, particularly the flavonoids, glycosides, and alkaloids, reduced pulmonary fibrosis [43]. Based on the clinical effectiveness of TCM, potential target discovery for asthma treatment is also a feasible strategy [44]. More research is needed to clarify the unconfirmed chemical composition and regulatory mechanisms, conduct standard clinical trials, and evaluate the possible side effects of these TCM herbs and formulations. The use of constitutions as an outcome indicator in the treatment of pulmonary disease is worth future studies [45].

The yin-deficiency constitution is significantly correlated with hypertension and diabetes mellitus [46]. Our finding indicates women with a vegetarian diet had a lower risk for the yin-deficiency constitution. It implies reducing the chance of a yin-deficiency constitution through a vegetarian diet may lower the risk of cardiovascular disease or diabetes mellitus. Some cross-sectional studies also indicate the vegetarian diet is associated with a lower fasting insulin level, higher insulin sensitivity, higher serum concentrations of polyunsaturated, higher monosaturated fatty acids, lower saturated fatty acids, lower long chain omega-3, lower trans fatty acids, and better renal function [47–49]. The vegetarian diet is also negatively associated with diabetes and impaired fasting glucose in premenopausal women and in menopausal women [50]. The experimental and longitudinal studies are suggested to explore the causal relationship between the yin-deficiency constitution and cardiovascular disease or diabetes mellitus among vegetarians, especially for indicators from blood tests. In addition, our result shows women with a high-school education or above have a higher risk of yang-deficiency constitution compared to those with elementary-school education. A survey indicates a significantly positive correlation existed between the yang-deficiency constitution and favor for hot food, and a negative correlation existed between regular exercise yang-deficiency constitution [51]. Collecting more data, such as different habits of diet and exercise, in middle-aged women, may be helpful to clarify the relationship between education level and yang-deficiency constitution.

Although we recruited participants from the Taiwan Biobank, our study did not include all possible participants because of the limited budget. Randomized sampling might have reduced potential selection bias. Women who did not participate in the Taiwan Biobank Survey might differ from those who took part in the survey and became a member of Taiwan Biobank. Hence, our results may not be generalizable outside of the Taiwan Biobank participants. Additionally, our findings are based on the analysis of secondary data. The secondary data analysis with a cross-sectional survey in our study could not provide the causality effect. Causality between variables and their associated constitutions becomes imperative for future prospective studies. Though BCQ revealed acceptable reliability and validity in previous studies [32–36], whether measuring the change of constitutions from longitudinal studies was consistent with the result in our study merits further study. With a stratified analysis of age, our findings show that there is no significant relationship between menstruation and deficiency/stasis constitution. It may be attributed to the lack of interaction between age and menstruation on deficiency/stasis constitution or small sample size in our study. The measurement with cutoff points of three constitutions in BCQ was applied in previous research [52–54] and our study. The method may be underestimating the change of constitutions. We measured only the deficiencies of yin and yang and stasis constitutions in middle-age women, and the study of more constitutions to measure women's overall health status are suggested for future studies.

## 5. Conclusion

Yin deficiency, yang deficiency, and stasis were common in middle-age women in Taiwan. Women with abnormal spirometry have had a higher risk for the constitutions in yin and yang deficiencies and stasis. Participants with current menstruation had a greater risk for a yang-deficiency constitution, while those younger than 56 years had a higher risk of yin-deficiency and stasis constitution. TCM doctors should focus their treatment on simultaneously balancing both deficiency and stasis constitutions. Future study is needed to examine the changes in deficiency and stasis constitutions among patients with climacteric symptoms or respiratory-related diseases or both.

## Figures and Tables

**Table 1:** Differences in demographic data and health-related variables between women with and without yang deficiency, yin deficiency, and stasis (*n* = 1000).

Variable	Total		Constitution					
			Yang deficiency (*n* = 297)		Yin deficiency (*n* = 217)		Stasis (*n* = 177)	
	*n*	(%)	*N*	(%)	*n*	(%)	*n*	(%)
*Age (years)*								
40 ≤ age < 51	419	(41.9)	142	(47.8)^*∗*^	102	(47.0)^*∗*^	99	(55.9)^*∗∗*^
51 ≤ age < 56	237	(23.7)	72	(24.2)	59	(27.2)	45	(25.4)
56 ≤ age < 65	344	(34.4)	83	(27.9)	56	(25.8)	33	(18.6)

*Marital status*								
Single	86	(8.6)	34	(11.5)^*∗∗*^	21	(9.8)	22	(12.4)^*∗∗*^
Married	744	(74.5)	206	(69.8)	152	(70.7)	117	(66.1)
Widowed	95	(9.5)	38	(12.9)	28	(13.0)	27	(15.3)
Divorced	73	(7.3)	17	(5.8)	14	(6.5)	11	(6.2)

*Currently working*								
No	419	(41.9)	114	(38.4)	83	(38.2)	59	(33.3)^*∗*^
Yes	581	(58.1)	183	(61.6)	134	(61.8)	118	(66.7)

*Education*								
Elementary school	60	(6.0)	8	(2.7)^*∗*^	10	(4.6)	4	(2.3)
High school	464	(46.4)	135	(45.5)	89	(41.0)	79	(44.6)
College or university	414	(41.4)	134	(45.1)	105	(48.4)	83	(46.9)
Graduate school	62	(6.2)	20	(6.7)	13	(6.0)	11	(6.2)

*Vegetarian*								
No	923	(92.3)	271	(91.2)	211	(97.2)^*∗∗*^	169	(95.5)
Yes	77	(7.7)	26	(8.8)	6	(2.8)	8	(4.5)

*Tea habit*								
No	709	(70.9)	204	(68.7)	150	(69.1)	122	(68.9)
Yes	291	(29.1)	93	(31.3)	67	(30.9)	55	(31.1)

*Coffee habit*								
No	661	(66.1)	181	(60.9)^*∗*^	131	(60.4)^*∗*^	101	(57.1)^*∗∗*^
Yes	339	(33.9)	116	(39.1)	86	(39.6)	76	(42.9)

*Menstruation*								
No	555	(55.5)	144	(48.5)^*∗∗*^	106	(48.8)^*∗*^	70	(39.5)^*∗∗*^
Yes	445	(44.5)	153	(51.5)	111	(51.2)	107	(60.5)

*Ever pregnant*								
No	101	(10.1)	38	(12.8)^*∗*^	23	(10.6)	21	(11.9)
Yes	899	(89.9)	259	(87.2)	194	(89.4)	156	(88.1)

*Ever abortion*								
No	432	(43.2)	124	(41.8)	85	(39.2)	71	(40.1)
Yes	568	(56.8)	173	(58.2)	132	(60.8)	106	(59.9)

*Body mass index*								
Underweight	21	(2.1)	7	(2.4)	5	(2.3)	4	(2.3)
Normal weight	595	(59.5)	181	(60.9)	133	(61.3)	98	(55.4)
Overweight	244	(24.4)	72	(24.2)	49	(22.6)	48	(27.1)
Obese	140	(14)	37	(12.5)	30	(13.8)	27	(15.3)

*Bone mineral density T score*								
Normal (≥1.0)	659	(65.9)	204	(68.7)	154	(71.0)	124	(70.1)
Osteopenia (−2.4 to−1.1)	267	(26.7)	74	(24.9)	52	(24.0)	45	(25.4)
Osteoporosis (≤−2.5)	74	(7.4)	19	(6.4)	11	(5.1)	8	(4.5)

*Bone mineral density Z score*								
Normal (≥−1.0)	909	(90.9)	273	(91.9)	203	(93.5)^*∗*^	163	(92.1)
Osteopenia (−2.4 to−1.1)	88	(8.8)	22	(7.4)	12	(5.5)	13	(7.3)
Osteoporosis (≤−2.5)	3	(0.3)	2	(0.7)	2	(0.9)	1	(0.6)

*Pulmonary function*								
Normal spirometry	600	(60)	164	(55.2)^*∗*^	117	(53.9)^*∗*^	93	(52.5)^*∗*^
Abnormal spirometry^*a*^	400	(40)	133	(44.8)	100	(46.1)	84	(47.5)

Chi-square testing was used to determine the significance of differences in characteristics between the women who had yang-deficiency, yin-deficiency, or stasis constitutions or not; ^*∗*^the significance level was set at *P* < 0.05; ^*∗∗*^*P* < 0.01. ^*a*^Included obstructive impairment, restrictive impairment, and mixed impairment.

**Table 2:** Menstruation effect is divided by age groups on the yang-deficiency, yin-deficiency, and stasis constitutions.

	Constitution								
	Yang deficiency			Yin deficiency			Stasis		
	OR	(95% CI)	*P* value	OR	(95% CI)	*P* value	OR	(95% CI)	*P* value
*40* ≤ *age* < *51 (yr)*									
Menstruation^*a*^	1.24	(0.68–2.28)	0.49	0.89	(0.47–1.68)	0.71	1.34	(0.67–2.71)	0.41

*51* ≤ *age* < *56 (yr)*									
Menstruation^*a*^	1.30	(0.73–2.33)	0.37	1.39	(0.76–2.57)	0.29	1.43	(0.73–2.80)	0.29

*56* *age* < *65 (yr)*									
Menstruation^*a*^	1.05	(0.11–10.22)	0.97	1.73	(0.18.16.91)	0.64	3.21	(0.32–31.75)	0.32

Analysis by logistic regression. *P* < 0.05 being statistically significant. OR: odds ratio. CI: confidence interval. Reference group: ^*a*^menopause.

**Table 3:** Logistic regression model results in yang-deficiency, yin-deficiency, and stasis constitutions.

	*B*	S.E.	Wald	*P* value	OR	95% CI
*Yang deficiency*						
Abnormal spirometry^*a*^	0.35	0.14	5.97	0.02	1.42	1.07–1.88
Menstruation^*b*^	0.37	0.14	6.85	<0.01	1.45	1.10–1.93
High school^*c*^	0.89	0.40	4.98	0.03	2.42	1.11–5.27
College or university^*c*^	1.03	0.40	6.67	0.01	2.80	1.28–6.12
Graduate school^*c*^	1.01	0.48	4.53	0.03	2.75	1.08–6.97

*Yin deficiency*						
40 ≤ age < 51 (yr)^*d*^	0.59	0.19	9.85	<0.01	1.81	1.25–2.62
51 ≤ age < 56 (yr)^*d*^	0.59	0.21	7.65	<0.01	1.80	1.19–2.72
Abnormal spirometry^*a*^	0.39	0.16	6.02	0.01	1.48	1.08–2.01
Vegetarian^*e*^	−1.29	0.43	8.78	<0.01	0.28	0.12–0.65

*Stasis*						
40 ≤ age < 51 (yr)^*d*^	1.13	0.22	26.18	<0.01	3.08	2.00–4.75
51 ≤ age < 56 (yr)^*d*^	0.83	0.25	11.04	<0.01	2.29	1.41–3.74
Abnormal spirometry^*a*^	0.49	0.17	8.08	<0.01	1.63	1.16–2.28
Coffee habit^*f*^	0.40	0.17	5.26	0.02	1.49	1.06–2.08

Multivariate logistic regression analysis was performed. *P* < 0.05 being statistically significant. OR: odds ratio. CI: confidence interval. Reference group:^*a*^normal spirometry; ^*b*^menopause; ^*c*^elementary school; ^*d*^56 ≤ age < 65 (yr.); ^*e*^not vegetarian;^*f*^without coffee habit.

**Table 4:** Items and identifications of yang-deficiency, yin-deficiency, and stasis constitutions in body constitution questionnaire (BCQ).

Constitution	Total items	Item number	Cutoff score
Yang deficiency	19	3, 5, 8, 9, 15, 16, 17, 22, 23, 24, 28, 31, 33, 36, 37, 41, 42, 43, 44.	30.5
Yin deficiency	19	2, 4, 8, 10, 11, 16, 18, 20, 23, 26, 29, 30, 31, 32, 35, 37, 38, 39, 40.	29.5
Stasis	16	1, 4, 5, 6, 7, 12, 13, 14, 16, 17, 19, 20, 21, 25, 27, 34.	26.5

## Data Availability

The datasets used and/or analyzed during the current study are available from the corresponding author on reasonable request.

## Appendix

Identifications of yang-deficiency, yin-deficiency, and stasis constitutions in body constitution questionnaire (BCQ): see Table 4.

References from:

1. L. L. Chen, J. S. Lin, J. D. Lin, et al., “BCQ+: a body constitution questionnaire to assess Yang-Xu. Part II: evaluation of reliability and validity,” *Forsch Komplementmed*, vol. 16, no. 1, pp. 20–27, 2009. doi: 10.1159/000197770.
2. J. S. Lin, L. L. Chen, J. D. Lin, et al., “BCQ−: a body constitution questionnaire to assess Yin-Xu Part II: evaluation of reliability and validity,” *Forschende Komplementärmedizin/Research in Complementary Medicine*, vol. 19, no. 6, pp. 285–292, 2012. doi: 10.1159/000346060.
3. J. D. Lin, J. S. Lin, L. L. Chen, et al., “BCQs: a body constitution questionnaire to assess stasis in traditional Chinese medicine,” *European Journal of Integrative Medicine*, vol. 4, no. 4, pp. e379e391, 2012. doi: 10.1016/j.eujim.2012.05.001.
4. C. H. Lee, T. C. Li, C. I. Tsai, et al., “Association between albuminuria and different body constitution in type 2 diabetes patients: Taichung diabetic body constitution study,” *Evidence-Based Complementary and Alternative Medicine*, vol. 2015, Article ID 603048, 8 pages, 2015. doi: 10.1155/2015/603048.
5. C. H. Lee, T. C. Li, C. I. Tsai, et al., “Yang deficiency body constitution acts as a predictor of diabetic retinopathy in patients with type 2 diabetes: Taichung diabetic body constitution study,” *Evidence-Based Complementary and Alternative Medicine*, vol. 2015, Article ID 940898, 8 pages, 2015. doi: 10.1155/2015/940898.
